# Opinion: the nature of primary and secondary synovial chondromatosis: importance of pathological findings

**DOI:** 10.3389/fonc.2023.1281890

**Published:** 2023-12-21

**Authors:** Jiro Ichikawa, Hiroki Imada, Tomonori Kawasaki, Hirotaka Haro

**Affiliations:** ^1^ Department of Orthopaedic Surgery, Interdisciplinary Graduate School of Medicine, University of Yamanashi, Yamanashi, Japan; ^2^ Department of Pathology, Saitama Medical Center, Saitama Medical University, Kawagoe, Japan; ^3^ Department of Pathology, Saitama Medical University International Medical Center, Saitama, Japan

**Keywords:** synovial chondromatosis, primary, secondary, pathology, etiology, molecular confirmation

## 1 Introduction

Synovial chondromatosis (SC) is a rare, benign tumor that frequently occurs in large joints and intra-articular areas such as the knee and hip (1). However, SC in the hands is uncommon, with the extra-articular (tenosynovial) type being more prevalent than the intra-articular type (1, 2). Imaging characteristics of SC include calcification observed on radiography and computed tomography and cartilaginous tissue observed on magnetic resonance imaging (2). SC has been categorized as primary or secondary depending on the onset factors and pathological findings (2, 3). Secondary SC is currently given descriptive names such as multiple osteochondral loose bodies (1). Senesi et al. reported a rare case of distal interphalangeal chondromatosis in the middle finger. Their report was published in *Anticancer Research* (4).

## 2 Subsections relevant to the subject

In their report, Senesi et al. concluded that their case was of “primary” SC (4). However, we find the classification into “primary” or “secondary” for their case to be debatable. This report reconsiders the distinctions between “primary” and “secondary” SC based on etiology, pathology, and prevalence.

## 3 Discussion

### 3.1 Etiology

Secondary SC may result from single or repeated mechanical stimulation (2). The factors contributing to secondary SC include trauma, such as fractures, dislocations, meniscus tears, sprains, osteochondritis dissecans, and osteoarthritis (OA) (3). In the case described by Senesi et al., intraoperative and postoperative radiography showed narrowing of the joint space, osteophyte formation, and dislocation in the distal interphalangeal (DIP) joints of the middle, index, and ring fingers. If this were a case of primary SC, the changes observed in the DIP joint of the middle finger could be attributed to SC progression. However, radiography findings for SC typically reveal calcification, while osteopenia and erosion are more indicative of primary OA (2). In addition, infectious and inflammatory diseases were excluded based on laboratory tests and the patient’s past medical history. Considering these imaging findings, this case appears to be of secondary SC induced by primary OA.

### 3.2 Pathology

Next, we will discuss the pathological findings, which are crucial for diagnosis. In their manuscript, Senesi et al. presented Figures 1C, D, displaying well-circumscribed nodules of the cartilage and bone (4). The cartilage exhibited moderate cellularity with varying degrees of ossification, consistent with both primary and secondary SC. However, an examination of the histological findings revealed some discrepancies. Senesi et al.’s paper depicted loose bodies containing chunky materials that histologically resembled bone fragments. Although the authors described these materials as an ossified chondroid matrix, their picture did not show continuity with the chondroid matrix nor match the calcification pattern typically seen in primary synovial chondromatosis, as reported by Villacin et al. Therefore, a more plausible explanation is that the chondroid matrix surrounded the bone fragments or osteophytes. These findings support the diagnosis of secondary SC rather than primary SC (3).

Additionally, there were other distinguishing features in the pathological findings. Primary SC exhibits a higher presence of binucleated cells and plump chondrocytes than does secondary SC. Furthermore, differences in the pattern of calcification and enchondral ossification were observed. These pathological findings, including binucleated cells, plump chondrocytes, and the pattern of calcification, did not allow strict differentiation between primary and secondary SC.

Recently, the classification of “secondary” SC is not often used. It is recognized as multiple osteochondral loose bodies because primary SC is considered a neoplastic change with rearrangement of FN1-ACVR2A and ACVR2A-FN1 fusion (1). In addition, especially in the case of patients with degenerative joint diseases like Senesi’s case, the histological findings could overlap due to the secondary modification, and detection of FN1 rearrangement by fluorescence *in-situ* hybridization or polymerase chain reaction would be valuable for correct diagnosis (1). Caution is warranted since i) *FN1* and *ACVR2A* rearrangements in SC are reportedly present in only 57% of cases, and ii) these rearrangements were reported in 75% of cases with chondrosarcoma secondary to SC (5). A recently recognized entity, designated calcified chondroid mesenchymal neoplasm (CCMN) (6), is also a potential differential diagnosis, and it has novel fusion genes, including FN1-MERTK, FN1-NTRK1, and FN1-TEK. The development of genetic analysis will unravel the nature of SC.

### 3.3 Clinical features

Villacin et al. analyzed 136 cases of SC, classifying 10 as primary SC and 126 as secondary SC (3). Of the 126 secondary cases, 10 had no clinical history or radiological findings, and only 10 (7%) were classified as primary SC. Furthermore, in a study focusing on SC in the shoulder, 9 of 10 cases (90%) were identified as secondary SC, and there were no definitive findings regarding clinical history, imaging, and operative findings in some cases (7). In both papers, one key limitation was the diagnosis using only pathology without molecular confirmation. The clinical course of primary SC seems more aggressive than that of secondary SC based on the recurrence rate (3, 7), which seems to reflect that secondary SC is not a neoplasm. While the rate of malignant transformation was small, approximately 5%–10%, there was a possibility of primary SC with large tumors and longstanding and multiple recurrences (1). Considering these clinical courses is valuable in correctly diagnosing primary SC, not secondary SC or multiple osteochondral loose bodies.

### 3.4 Conclusion

Herein, we have described in detail the current concept of SC. Primary SC is simply a neoplasm, and secondary SC and multiple osteochondral loose bodies, a manifestation of degenerative joint disease, must be distinguished from primary SC. Based on clinical courses and pathological findings, it can be difficult to distinguish between primary SC and secondary SC/multiple osteochondral loose bodies. Molecular confirmation of FN1 and ACVR2A rearrangements by FISH may serve as a useful ancillary tool for confirming primary SC. Because clinical features, including malignant transformation and recurrence, differ markedly between these two disease entities, a correct diagnosis is essential.

## Author contributions

JI: Writing – original draft, Writing – review & editing. HI: Writing – original draft, Writing – review & editing. TK: Writing – original draft, Writing – review & editing. HH: Writing – original draft, Writing – review & editing.
